# Case report: Isolated oligometastatic disease of the prostate from a primary lung adenocarcinoma

**DOI:** 10.3389/fonc.2024.1394168

**Published:** 2024-05-22

**Authors:** Josette M. Kamel, Simran Arjani, Kateryna Fedorov, Fnu Sapna, Jinrong Cheng, Ioannis Mantzaris

**Affiliations:** ^1^ Department of Oncology, Montefiore Medical Center Albert Einstein College of Medicine, The Bronx, NY, United States; ^2^ Department of Pathology, Montefiore Medical Center Albert Einstein College of Medicine, The Bronx, NY, United States

**Keywords:** prostate cancer, lung adenocarcinoma, tumor to tumor metastasis, secondary prostate cancer, lung adenocarcinoma with oligometastatic disease

## Abstract

Secondary prostate cancer typically occurs from direct seeding of a renal or bladder tumor. Metastasis via hematogenous spread is exceedingly rare and is typically identified incidentally at autopsy. This report describes a 72-year-old male with lung adenocarcinoma initially staged as Stage IA2 who developed oligometastatic disease of the prostate. He was initially treated with radiation therapy and was found to have a hypermetabolic focus in the prostate gland during surveillance PET/CT imaging 6 months following treatment. Subsequent biopsy revealed metastatic lung adenocarcinoma in 6/6 core samples, leading to diagnosis of oligometastatic disease of the prostate. To our knowledge, this is the first report of isolated oligometastatic disease to the prostate from a primary lung adenocarcinoma.

## 1 Introduction

**Figure 1:**
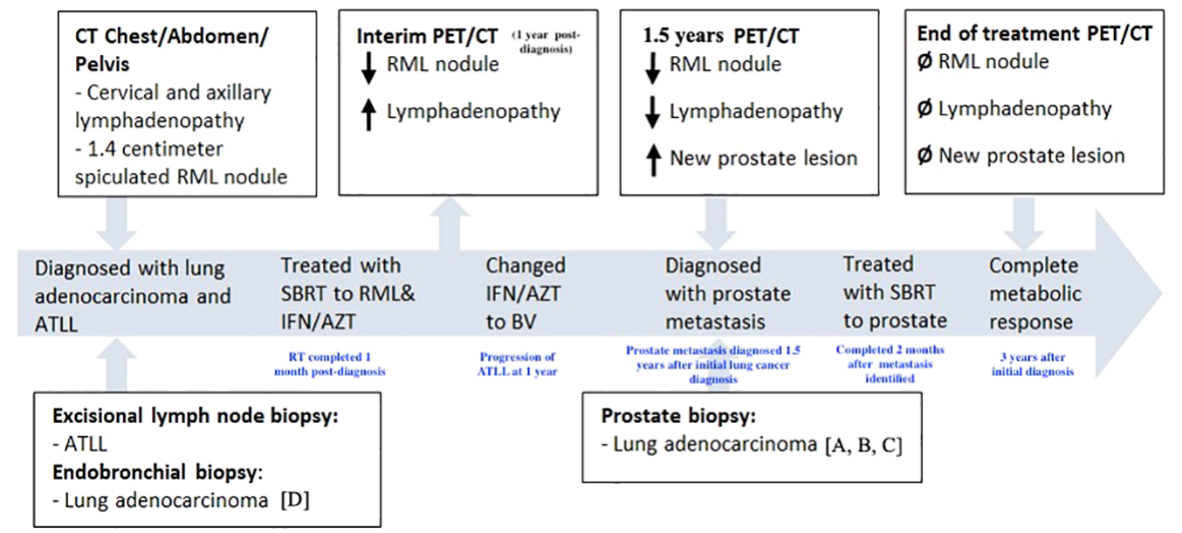
Timeline of treatment course. CT, computed tomography. PET, positron emission tomography. RML, right middle lobe. ATLL, adult T-cell leukemia/lymphoma. SBRT, stereotactic body radiation therapy. IFN/AZT, interferon/zidovudine. BV, brentuximab vedotin. Letters presented in brackets (ex: [A]) refer to parts of pathology shown in **Figure 3**.

Prostatic metastasis is uncommon, occurring in less than one percent of all prostatic surgical specimens (1). Leukemia and lymphoma are the most common neoplasms to metastasize to the prostate (1). Solid tumor metastases typically occurs by direct extension from nearby organs and is most commonly seen in patients with widely disseminated metastatic disease (2). Hematogenous spread is extremely rare, and only a few cases have been documented, including metastasis from primary lung, gastrointestinal, skin and endocrine organs (1). Due to their propensity to be identified at later stages, and typically in widely disseminated disease, these malignancies commonly confer poor prognoses and are typically identified incidentally during autopsy (2). In this report, we describe an unusual case of isolated oligometastatic disease of the prostate from a primary lung adenocarcinoma (**Figure 1**).

## 2 Case presentation

**Figure 2:**
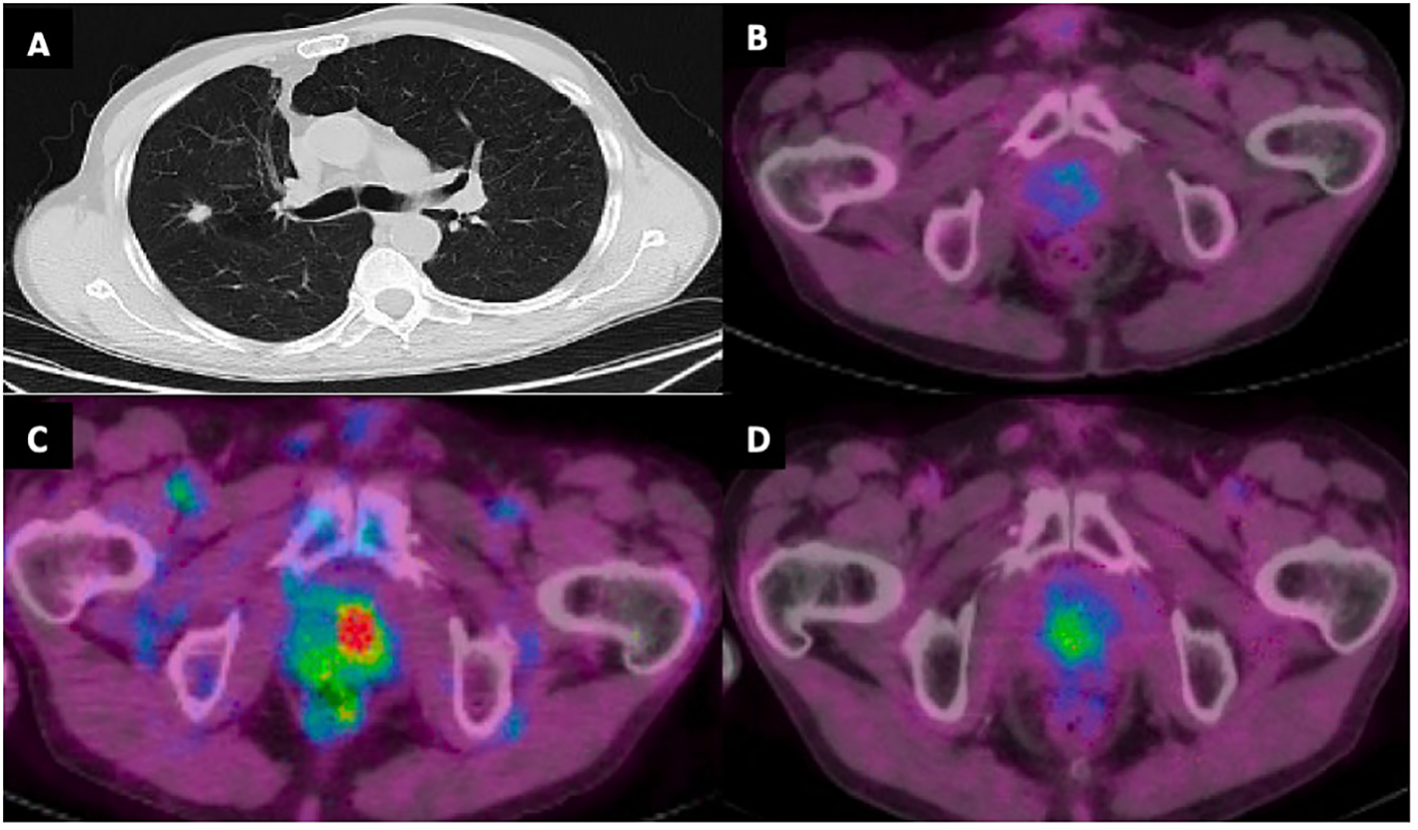
**(A)** CT chest with 1.4 cm right middle lobe spiculated nodule at time of initial diagnosis. **(B)** PET/CT demonstrating absence of increased FDG activity in the prostate gland at time of diagnosis. **(C)** Increased FDG activity in the prostate gland with maximum standardized uptake value (SUV) 8.4 concerning for prostatic metastasis. **(D)** Decreased FDG activity in prostate gland following SBRT with SUV max 3.6.

A 72-year-old male with a 20-pack-year smoking history, presented with a painless right neck mass and axillary lymphadenopathy. Imaging of the neck showed multiple slightly enlarged right cervical nodes and right axillary nodes, as well as a 1.4 cm right middle lobe (RML) spiculated nodule (**Figure 2A**). PET/CT demonstrated absence of increased signal activity in other organs, including the prostate gland (**Figure 2B**). Excisional biopsy of a right neck lymph node revealed mature CD4+, CD7-, CD8-, CD25+, CD30+ T- cell lymphoma. He tested positive for human-T-lymphocytic virus type 1 (HTLV-1) and was diagnosed with Adult T Cell Leukemia/Lymphoma (ATLL). Staging positron emission tomography/computed tomography (PET)/CT did not reveal additional sites of disease. There was no bone marrow involvement. The patient underwent endobronchial ultrasound-guided biopsy of the spiculated RML pulmonary nodule, which revealed lung adenocarcinoma. There was no nodal involvement and it was classified as a Stage IA2 (cT1bN0M0) tumor. Molecular testing revealed EGFR-negative status and PD-L1 TPS < 1%. It was treated with 54 Gy of stereotactic body radiation therapy (SBRT). The patient’s ATLL was treated with Interferon and Zidovudine. Repeat PET/CT showed interval decrease of RML nodule but progression of ATLL prompting change of treatment to brentuximab vedotin (BV). Repeat PET/CT 6 months later showed resolution of lymphadenopathy and previously seen RML nodule but identified an ill-defined right lower lobe (RLL) opacity attributed to post-radiation changes.

**Figure 3:**
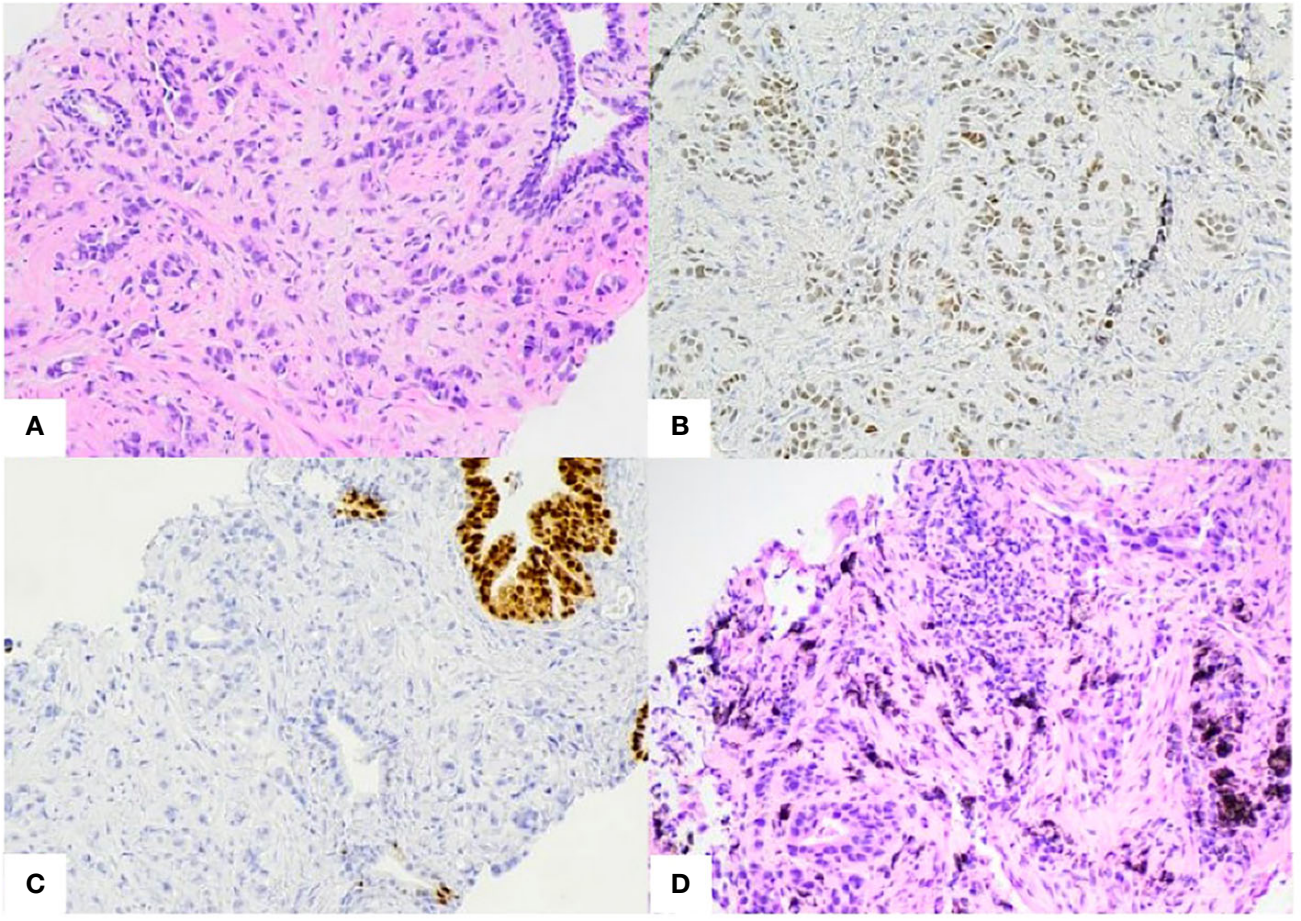
**(A)** Prostate biopsy. Poorly differentiated pulmonary adenocarcinoma infiltrating between benign prostate glands. **(B, C)**. Prostate biopsy immunohistochemistry stains. Positive for TTF1 [B], CK7, AE1/3 (not shown) and negative for NKX3.1[C] and Napsin-A (not shown). **(D)** Right middle lobe biopsy. Invasive pulmonary adenocarcinoma. Positive for TTF-1, Napsin-1, and negative for p40 (not shown).

Subsequent PET/CT 1.5 years following initial diagnosis revealed interval enlargement of the RLL opacity consistent with post-radiation changes and a new hypermetabolic focus in the left prostate concerning for malignant process at both locations (**Figure 2C**). Prostate Specific Antigen (PSA) testing was negative. A prostate biopsy identified infiltration of prostatic gland by a poorly differentiated adenocarcinoma in 6/6 core samples. Tumor cells were positive for TTF1, CK7, AE1/3 and negative for NKX3, suggestive of metastatic lung adenocarcinoma (**Figure 3**). Given oligometastatic, low burden disease he was treated with SBRT at both sites. He received 45 Gy and 24 Gy at the RLL and prostate, respectively. He completed 16 months of BV with continuous complete metabolic response of his lymphoma on post-treatment PET/CT (**Figure 2D**). At the time of the writing of this report the patient has been in remission for one year.

## 3 Discussion

Over half of lung adenocarcinoma cases are metastatic at diagnosis, commonly involving the liver, adrenal glands, bone, and brain (3). Lung cancer metastasis to prostate is uncommon, with most cases found incidentally during autopsy (1). A study reporting 1,474 autopsy cases of prostate tumors identified only 18 (1.2%) cases with metastatic prostatic lesions;5 (0.34%) cases were derived from primary lung malignancies (4). In a series of approximately 30,000 patients with metastatic cancer, a diagnosis of metastasis to the prostate was made in 3 (0.01%) of cases (4). Another study at the Royal London Hospital examined post-mortem samples from 11,945 prostatic resections and biopsies, obtained from 1907 to 2002. Of these, 51 secondary prostatic tumors were identified, including eight that originated from primary lung cancers. All of these patients represented had widely disseminated disease and not isolated metastasis to the prostate (1). A post-mortem pathological examination of 10,791 cancer patients at the Roswell Park Comprehensive Cancer Center in New York also identified 185 cases of secondary neoplasms of the prostate. In 181 patients tumor was present in at least five or more organ systems. Only one of the four remaining cases involved a primary lung malignancy and this patient may have had disease in multiple sites as well (5). These cases of prostate metastasis were identified at autopsy, not during the patient’s active clinical management, and they occurred almost exclusively in the context of widely disseminated disease. This contrasts with our patient, who was diagnosed during ongoing clinical treatment and exhibited only isolated oligometastasis to the prostate, without evidence of disease at additional sites.

A PubMed search for cases of prostatic tumors composed of a primary lung cancer revealed 6 cases (**Table 1**) (2, 3, 6–9). Disseminated disease was present in five cases; one report did not specify. In the majority of cases the PSA was not significantly elevated, and patients had lower urinary tract symptoms such as hematuria, pelvic pain, and the sequelae of urethral obstruction (1). These symptoms are associated with benign prostatic hyperplasia, primary prostate tumors, and secondary prostate tumors. Because the symptoms of primary and secondary neoplasms of the prostate overlap it is impossible to distinguish them cinically (1).

**Table 1:** Case reports documenting secondary disease of the prostate from primary lung cancer.

Author	Year	Age	Primary Lung Tumor	Symptoms	PSA (ng/ml)	Additional sites of metastasis	Histology	Treatment
Smedley (6)	1983	48y	Small cell^a^	Dysuria and hesitancy	NR	Liver, peritoneal and para-aortic LN	NR	Radical RT
Ohmori (7)	1998	81y	Small cell	Dysuria	62.5	Kidney, bladder, multiple LN	NR	Hormonal therapy, visual laser ablation
Gonzalez Yañez (3)	2009	64y	Small cell	Back pain	14.9	Adrenal glands, kidneys, pancreas, thoracic vertebrae^b^	NR	RT
Yoo (2)	2009	70y	Neuroendocrine large cell carcinoma	Difficulty voiding	NR	Brain, bone, liver, LN	Positive: SPY, CgA, CK7, TTF-1. Negative: PSAP, P504S/AMACR, PSA, CK20, CD56, and p63	TURP
Barba Abad (8)	2010	82y	Mixed adenocarcinoma and squamous cell carcinoma	Refractory urinary tract infection	4.4	Unknown^c^	Positive: CK 7 Negative: PSA, p63, uroplakin-III	TURP
Gilmour (9)	2019	55y	Adenocarcinoma	Weakness, moderate pelvic pain, difficult urination	6.8	Brain, bone, adrenal glands	Positive: TTF-1 Negative: PSA	Palliative RT

Key. y, years. NR, not reported. LN, lymph nodes. RT, radiation therapy. TURP, Transurethral Resection of the Prostate. SPY, synaptophysin. CgA, chromogranin A. CK7, cytokeratin 7. TTF-1, Thyroid transcription factor–1. AMACR, alpha methylacyl CoA racemase.

a identified following prostate cancer diagnosis.

b Identified one year following diagnosis of prostate cancer.

c Patient was found to have pulmonary progression at time of prostate cancer diagnosis and passed one month.

Differentiating between primary and secondary disease of the prostate by close histological examination is critical, as hormonal treatments and surgical excision may not be indicated for the latter. Primary prostate cancers have highly variable microscopic features (10). Secondary neoplasms of the prostate typically have well-circumscribed appearance on pathology, tumor invasion to the non-neoplastic glands, and absence of intraepithelial neoplasia in adjacent glands (4). To further delineate tissue of origin, immunohistochemistry for tissue-specific markers is informative. Secondary prostatic tumors are likely negative for PSA and Prostatic Acid Phosphatase (PAP), as these enzyme secretions are intrinsic and specific to the prostate gland (4). Secondary lung adenocarcinomas typically overexpresses CK7, TTF-1, and AE1/3. TTF-1, in particular, is highly sensitive and specific for primary lung adenocarcinoma and can be reliably used to differentiate between metastatic lung disease and primary prostatic tumors (3). Prostate adenocarcinoma typically stains positive for NKX3.1 and Napsin-A, although the latter is also frequently positive in lung adenocarcinoma as well. And while it is not yet standard of care recent research suggests that RNA expression of prostate cancer antigen 3 (PSA3) is specific to primary prostate tumors, and may represent a valuable tool as a cancer-specific biomarker in distinguishing primary from secondary disease (9, 11–13).

## 4 Conclusion

The prostate is a rare but possible site of metastasis of primary lung adenocarcinoma. To our knowledge, this is the first case of biopsy-proven lung adenocarcinoma with isolated oligometastatic disease to the prostate emphasizing the importance of paying close attention to and pursuing workup of all hypermetabolic lesions identified on PET/CT. Prompt identification of tissue origin allows for timely and appropriately tailored therapeutic intervention.

## Data availability statement

The raw data supporting the conclusions of this article will be made available by the authors, without undue reservation.

## Ethics statement

Written informed consent was obtained from the individual(s) for the publication of any potentially identifiable images or data included in this article.

## Author contributions

JK: Data curation, Writing – original draft, Writing – review & editing. SA: Writing – original draft, Writing – review & editing. KF: Conceptualization, Writing – original draft, Writing – review & editing. FS: Data curation, Writing – original draft, Writing – review & editing. JC: Data curation, Writing – review & editing. IM: Conceptualization, Data curation, Resources, Supervision, Writing – original draft, Writing – review & editing.
